# Identification of a Novel KPC Variant, KPC-204, Conferring Resistance to Both Carbapenems and Ceftazidime–Avibactam in an ST11 *Klebsiella pneumoniae* Strain

**DOI:** 10.3390/microorganisms12061193

**Published:** 2024-06-13

**Authors:** Yanqiao Gong, Yu Feng, Xiaoju Lv

**Affiliations:** 1 Center of Infectious Diseases, West China Hospital, Sichuan University, Chengdu 610041, China; gongyq@hbmzu.edu.cn (Y.G.); feng_yu@scu.edu.cn (Y.F.); 2 Department of Infection Control, Minda Hospital, Hubei Minzu University, Enshi 445000, China; 3 Center for Pathogen Research, West China Hospital, Sichuan University, Chengdu 610041, China; 4 Division of Infectious Diseases, State Key Laboratory of Biotherapy, Chengdu 610041, China

**Keywords:** KPC-204, ceftazidime–avibactam, *Klebsiella pneumoniae*

## Abstract

This study describes KPC-204, a novel variant of *Klebsiella pneumoniae* carbapenemase, characterized by a Lys-Asp-Asp (KDD) amino acid insertion at Ambler position 269 deviates from KPC-2. This variant was identified in an ST11-type clinical isolate of carbapenem-resistant *Klebsiella pneumoniae* from China. Notably, KPC-204 exhibits resistance to both ceftazidime-avibactam and carbapenems. Genetic analysis revealed that *bla*_KPC-204_ was located on a highly mobile IncFII/IncR plasmid within a complex genetic structure that facilitates its spread. Functional analysis, achieved through cloning into *E. coli* DH5α, validates KPC-204’s contribution to increased resistance to ceftazidime-avibactam. The kinetic parameters showed that KPC-204 exhibited similar affinity to KPC-2 toward ceftazidime and reduced sensitivity to avibactam. Docking simulations revealed a weaker interaction between KPC-204 and avibactam compared to KPC-2. Mating experiments demonstrated the resistance’s transmissibility. This investigation underscores the evolving diversity of KPC variants affecting ceftazidime-avibactam resistance, highlighting the necessity for continuous monitoring.

## 1. Introduction

Carbapenem-resistant *Klebsiella pneumoniae* (CRKP) with the *Klebsiella pneumoniae* carbapenemase (KPC) gene is a significant concern worldwide [1]. KPC enzymes degrade β-lactam antibiotics, including carbapenems, and are resistant to conventional β-lactamase inhibitors. Ceftazidime-avibactam (CZA) has been crucial for its effectiveness and safety in treating the infections caused by carbapenem-resistant *Enterobacteriaceae* (CRE) [2,3]. Nonetheless, the rise of CZA resistance among KPC-producing CRKP strains has become alarming, necessitating vigilant surveillance. The mechanisms of resistance to CZA in KPC-producing strains could be due to the coproduction of less sensitive β-lactamases, to changes in membrane permeability including loss or mutations in porins, or to efflux pumps [4]. Nevertheless, the most frequent mechanism remains the mutation in KPC-encoding genes [4,5]. Previously reported mutations like KPC-31/KPC-33(D179Y) [6,7], T243A (unassigned) [8], KPC-128 (D179Y/T243M) [8], KPC-134 (D178A with an insert sequence of aspartic acid–aspartic acid–asparagine–arginine–alanine–proline–asparagine–lysine) [9], KPC-93 (T237S and H274Y) [10], KPC-74 (G239_V240 deletion) [11], and KPC-71 (S182 insertion) [12], these genetic alterations are associated with increased CZA minimum inhibitory concentration (MIC) and decreased meropenem (MEM) MIC in comparison to wild-type isolates. The extension of resistance to CZA is associated with a trade-off in the lose resistance to carbapenem [4]. This balance is clinically beneficial, allowing for the combined use of CZA and carbapenems in treatment [13]. We identified a novel KPC variant, KPC-204, in an ST11 *K. pneumoniae* isolate from China. This variant contains a KDD insertion at Ambler position 269 within loop 267–275 [14], representing a mutational hotspot distinct from KPC-2. KPC-204 significantly decreases susceptibility to CZA, yet remains effective against carbapenems.

## 2. Materials and Methods

### 2.1. The Strains and In Vitro Susceptibility

The *K. pneumoniae* strain, designated 130125, was isolated from the respiratory tract secretion of a patient in 2017 in the intensive care unit (ICU) at West China Hospital. The patient received treatment with penicillin and meropenem for a duration of 12 days, followed by a 3-day course of cefoperazone–sulbactam before the sample was taken. Preliminary species identification was performed by Vitek II (bioMérieux, Marcy-l’Étoile, France). The MICs of antimicrobial agents were determined using the microdilution method of the Clinical and Laboratory Standards Institute (CLSI) [15]. Avibactam was added at a fixed concentration of 4 mg/L, tazobactam at 4 mg/L, relebactam at 4 mg/L, and vaborbactam at 8 mg/L.

### 2.2. Whole Genome Sequencing and Analysis

The genomic DNA of strain 130125 was prepared using the QIAamp DNA Mini Kit (Qiagen, Hilden, Germany) and sequenced using both HiSeq X10 (Illumina; San Diego, CA, USA) and MinION (Nanopore; Oxford, UK) platforms. Both short (Illumina) and long (Nanopore) reads were utilized to generate a de novo hybrid assembly using Unicycler v0.5.0 [16] under conservative mode and polished using Pilon v1.24 [17]. FastANI v1.33 [18] was used to calculate the pairwise average nucleotide identity (ANI) between 130125 and the type strain of *K. pneumoniae* (GCF_000240185) for precise species identification, with a cut-off of 96% applied to define a bacterial species [19]. The sequence type (ST) was determined by querying the PubMLST database [20] using MLST v2.23.0 (https://github.com/tseemann/mlst; accessed on 28 April 2024), while capsule (KL) and outer membrane porins were typed using Kleborate v2.3.2 [21]. Antimicrobial resistance genes and plasmid replicons were identified from the genome sequences using the ABRicate v1.0.0 (https://github.com/tseemann/abricate; accessed on 28 April 2024) to query the ResFinder database (http://genepi.food.dtu.dk/resfinder; accessed on 28 April 2024) and PlasmidFinder [22] database (accessed on 28 April 2024), respectively. Plasmid comparison was performed using BRIG v0.95 [23] in the default settings. Insertion sequences were identified using ISFinder (https://isfinder.biotoul.fr/; accessed on 28 April 2024) [24].

### 2.3. qRT-PCR

The expression of the *bla*_KPC_ gene was assessed using qRT-PCR. RNA was extracted from late-exponential-phase cultures using the TRIzol reagent (Invitrogen China Limited, Beijing, China). Genomic DNA was removed, and cDNA was synthesized using the PrimeScript RT Reagent Kit with gDNA Eraser (TaKaRa Biotechnology (Dalian), Dalian, China). qRT-PCR was conducted on a LightCycler 96 (Roche, Basel, Switzerland) using SYBR Premix Ex Taq II (Tli RNaseH plus kit) (TaKaRa Biotechnology (Dalian), Dalian, China) according to the manufacturer’s instructions. The assays utilized primers for *bla*_KPC_ and the housekeeping gene *rpoB* listed in Table S1. The expression of *bla*_KPC_ was normalized to the housekeeping gene *rpoB*. The relative expression was calibrated against isolate 015093. Relative transcript levels were calculated using the 2^−ΔΔCT^ formula based on the mean values. For each strain, three independent cultures were used to extract RNA as three biological replicates, and for each RNA sample, the whole process of qRT-PCR was repeated in triplicate as technical replicates.

### 2.4. Cloning Experiment

To evaluate the KPC-204 variant’s role in CZA resistance in *K. pneumoniae* 130125, the *bla*_KPC-204_ and *bla*_KPC-2_ genes, along with their promoter regions, were amplified from strains 130125 and 015093 using primers KPC_NdeI_F and KPC_EcoRI_R listed in Table S1. The amplified products and pET-28a vector were digested with NdeI and EcoRI enzymes, ligated with T4 ligase, and transformed into *E. coli* DH5α (Tsingke, Beijing, China), as described before [25]. Transformants were selected on Luria-Bertani agar plates containing 50 mg/L kanamycin, confirmed through PCR-employing primers KPC-F and KPC-R listed in Table S1, followed by Sanger sequencing validation. A control utilizing the empty vector pET-28a was similarly established in *E. coli* DH5α.

### 2.5. Kinetic Assay and Determination of IC_50_ Values

The *bla*_KPC_ gene sequence (residues 25–293) was cloned into the pET-28a vector using In-Fusion HD Cloning Kits (Takara Bio, Kusatsu, Japan). The *bla*_KPC_ gene sequence (residues 25–293) and the pET-28a vector were amplified with the primers listed in Table S1. The resultant recombinants were then transformed into *E. coli* Transetta (DE3)-competent cells (Novagen, Sacramento, CA, USA). Protein purification was achieved via Ni-NTA affinity chromatography, as described previously [26]. Extinction coefficients and wavelengths were adopted from earlier studies [11]. The enzyme kinetics assay was performed in triplicate using a SPECTROstar Nano microplate reader (BMG Labtech, Ortenberg, Germany) for 15 min in each round. Kinetic parameters (*K*_m_ and *k*_cat_) were determined using non-linear regression analysis with GraphPad Prism 9.0 (GraphPad Software, San Diego, CA, USA).

The IC_50_ values for the inhibition of KPC-2 and KPC-204 proteins by avibactam, tazobactam, and clavulanic acid were determined using nitrocefin as the substrate. The enzymes were mixed with these inhibitors at concentrations ranging from 0 to 30 μM in PBS and incubated for 10 min, after which 100 μM of nitrocefin was added. Absorbance at 482 nm was recorded after 30 min and analyzed with GraphPad Prism 9.0 (GraphPad Software, San Diego, CA, USA). This procedure was replicated in three independent experiments.

### 2.6. Structure Prediction

The crystal structure of the KPC-2 protein was retrieved from the PDB database (PDB ID: 2OV5, https://doi.org/10.2210/pdb2OV5/pdb, accessed on 28 April 2024) [27]. AlphaFold v2.2.3 [28] was employed to generate a structural model for the KPC-204 mutant. All software utilized, including AlphaFold v2.2.3 for CC312 modeling and the openMM Amber force field for protein relaxation, ran on a local server equipped with a powerful dual-channel E5-2697 Intel 24-core CPU, Nvidia A100 (40 GB) GPU, 96 GB of memory, for efficient computation. Notably, AlphaFold’s “monomer” model searched all genetic databases used in CASP14 to create the most accurate model, with the final selection based on the highest plddt confidence score. This combined approach ensured the availability of reliable protein structures for further analysis.

### 2.7. Comparative Secondary and Stereoscopic Structures

Amino acid sequence alignments and secondary structure predictions were conducted using ESPript 3 [29], with the crystal structure of KPC-2 (PDB ID: 2OV5) as a reference for secondary structure depiction. Stereoscopic structures of KPC-204 and KPC-2 were aligned using PyMOL v2.5.2 software.

### 2.8. Molecular Docking

AutoDock4.2.6 [30] software was used for the molecular docking simulations. Three-dimensional protein structures were retrieved from the Protein Data Bank (PDB) database and AlphaFold modeling. Ligand structures were built using Chem3D v20.1 software. The protein and ligand structures were then prepared for docking calculations by converting them into the pdbqt file format according to the AutoDock protocol. All docking parameters were maintained at their default settings, except for the maximum number of energy evaluations (eval) and the number of genetic algorithm (GA) runs. The docking simulations were executed using the prepareCovalent.py Python script, and the resulting interactions were analyzed using PyMOL software. A site-specific approach ensured ligands explored the relevant binding pocket of the KPC enzymes. The grid box, with a spacing of 0.375 Å and dimensions of 60 × 60 × 60 points, was centered at coordinates (59.28, −23.36, −4.18) Å.

### 2.9. Mating Experiments

Mating experiments were performed in broth and on filters, using *E. coli* J53 AziR (an azide-resistant variant of J53) as the recipient, at 25 °C and 37 °C, as described previously [31]. Transconjugants were selected on LB agar with 4 mg/L of ceftazidime and 150 mg/L of sodium azide. The *bla*_KPC-204_ gene and plasmid replicons in transconjugants were verified via PCR using primers listed in Table S1, followed by confirmation through Sanger sequencing.

## 3. Results

### 3.1. Antimicrobial Susceptibility

The *K. pneumoniae* isolate 130125 was resistant to piperacillin, piperacillin–tazobactam, cefoxitin, cefepime, ceftazidime, aztreonam, imipenem, meropenem, and ertapenem, but was susceptible to imepenem–relebactam and meropenem–vaborbactam (Table 1). Moreover, the isolate was resistant to CZA, with a MIC of 256 mg/L.

### 3.2. Genomic Analysis of Clinical K. pneumoniae Isolate 130125

The complete genome sequence of strain 130125 was obtained by de novo hybrid assembly of both short (Illumina) and long (Nanopore) reads, and had a 5.4 Mb circular chromosome and three plasmids (Table 2). Strain 130125 harbored the *bla*_KPC-204_ gene, encoding KPC-204, a novel variant with a three-amino-acid insertion (Lys-Asp-Asp) between amino acids 268 and 269 within loop 267–275, diverging from KPC-2. Strain 130125 was identified as *K. pneumoniae*, exhibiting 99.67% average nucleotide identity (ANI) with reference strain HS11286 (GCF_000240185.1), exceeding the classification threshold of ≥96% ANI for bacterial species. Strain 130125 was classified as ST11, a prevalent type of CRKP in China, and exhibited the KL64 capsule type. The genetic assessment of *ompK35* and *ompK36* genes identified characteristic mutations in ST11 *K. pneumoniae* strains included a truncation in *ompK35* and an insertion of GD amino acids at positions 134–135 in *ompK36* [32].

### 3.3. Genetic Context of bla_KPC-204_-Carrying Plasmid

**Figure 1:**
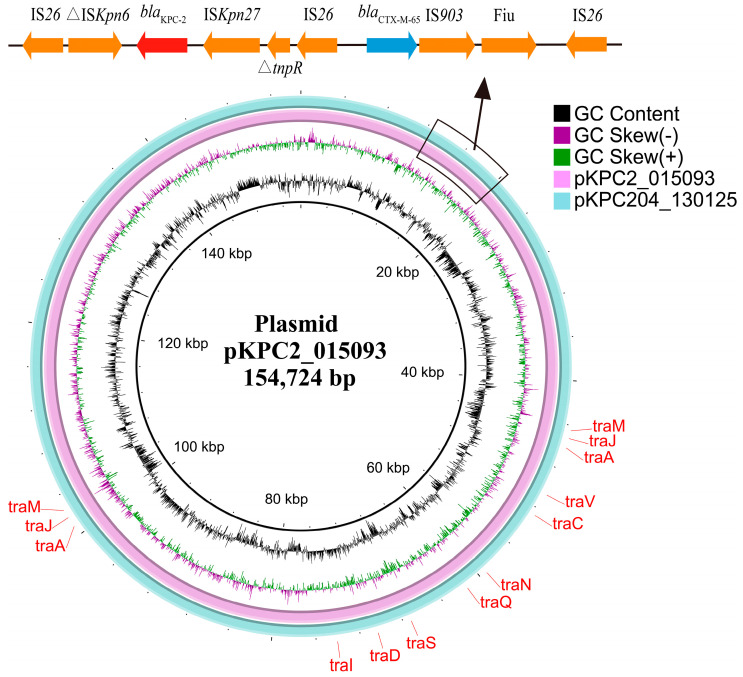
Alignment of pKPC204_130125 with pKPC2_015093. pKPC2_015093 is used as a reference. The alignment, conducted as a pairwise BLASTn comparison using the BLAST Ring Image Generator (BRIG) [23], between plasmid pKPC204_130125 and pKPC2_015093 (GenBank accession no. CP036301) demonstrated a 100% coverage and 99.97% identity. The *bla*_KPC-204_ gene is located within a composite transposon, flanked downstream by IS*Kpn27* and upstream by IS*Kpn6*. Moreover, *bla*_KPC-204_, in conjunction with *bla*_CTX-M-65_, is part of a 10 kb integrative composite transposon, bounded by IS*26* sequences. The locations of *tra* genes, pivotal for conjugation [34], are indicated.

Strain 130125 harbors genes for four β-lactamases including narrow-spectrum β-lactamases gene *bla*_SHV-187_ [33] on the chromosome. Additionally, the novel carbapenemase gene *bla*_KPC-204_, alongside *bla*_CTX-M-65_ and *bla*_TEM-1_, is located on a 154-kb IncFII/IncR plasmid, designated as pKPC204_130125 (Table 2). An alignment of pKPC204_130125 with pKPC2_015093 (GenBank accession no. CP036301) revealed a 100% coverage and 99.97% identity, indicating significant genetic similarity (Figure 1). The *bla*_KPC-204_ gene is located within a composite transposon, flanked downstream by IS*Kpn27* and upstream by IS*Kpn6*. Moreover, *bla*_KPC-204_, in conjunction with *bla*_CTX-M-65_, is part of a 10 kb integrative composite transposon, bounded by IS*26* sequences (Figure 1). This arrangement underscores the genetic mobility potential, facilitating the dissemination of antibiotic resistance.

### 3.4. Relative bla_KPC_ Gene’s Expression Levels

**Figure 2:**
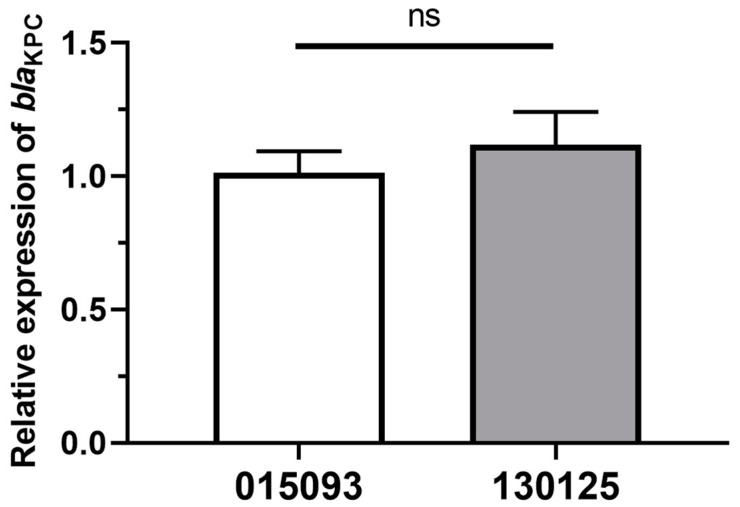
The relative expression level of *bla*_KPC_ compared to strain 015093. The relative expression of *bla*_KPC_ mRNA in isolate 130125 was 1.104 ± 0.135 times that of isolate 015093 (*p* > 0.05); ns, *p* > 0.05 (Student’s *t*-test).

Quantitative Real-Time PCR (qRT-PCR) was employed to assess the relative *bla*_KPC_ gene’s expression levels using *K. pneumoniae* 015093 [35] (an ST11 isolate that produces *bla*_KPC-2_ and exhibits susceptibility to CZA, sourced from our institution, with MIC values presented in Table 1) as a reference. The expression of *bla*_KPC_ in isolate 130125 showed a (1.104 ± 0.135)-fold increase compared to isolate 015093, a difference that was statistically insignificant (*p* > 0.05) (Figure 2).

### 3.5. Identification of bla_KPC-204_ Involved in CZA Resistance

*bla*_KPC-204_ and *bla*_KPC-2_ were successfully cloned into pET28a, generating pEKPC-204 and pEKPC-2. In *E. coli* DH5α, pEKPC-204 conferred resistance to a broad spectrum of β-lactams, notably to CZA (MIC 64/4 mg/L), as well as to imipenem, meropenem, and ertapenem (MICs 16 mg/L, 16 mg/L, and 8 mg/L, respectively), while remaining susceptible to imipenem–relebactam and meropenem–vaborbactam (MICs 0.125/4 mg/L and 0.125/8 mg/L, respectively). Remarkably, the CZA MIC for DH5α::pEKPC-204 was 128 times higher than that for DH5α::pEKPC-2 (Table 1). When tested with a fixed ceftazidime concentration of 2 mg/L, the MIC for avibactam against DH5α::pEKPC-204 was 16 mg/L, four times the MIC for DH5α::pEKPC-2 (4 mg/L), highlighting the significant elevation in avibactam resistance attributable to the KPC-204 variant.

### 3.6. Enzyme Kinetic Parameters and IC_50_ Values

The enzymatic kinetics analyses revealed that KPC-204 exhibits comparable catalytic efficiencies with substrates such as nitrocefin, ceftazidime, and meropenem to those of KPC-2, as shown in Table 3. The hydrolytic profile of KPC-204 was consistent with the MIC observations presented above (Table 1). Moreover, the IC_50_ value, defined as the concentration required to achieve 50% inhibition of avibactam, was approximately 16 times greater for KPC-204 compared to KPC-2. This suggests that the insertion at position 269 (ins_269_KDD) in KPC-204 correlates with a reduced affinity and diminished sensitivity to avibactam. In contrast, the inhibitory effects of tazobactam and clavulanic acid on KPC-204 were markedly stronger than on KPC-2, displaying approximately 21-fold and 7-fold lower IC_50_ values, respectively (Table 4).

### 3.7. Comparative Secondary and Stereoscopic Structures of KPC-204 and Related Variants

**Figure 3:**
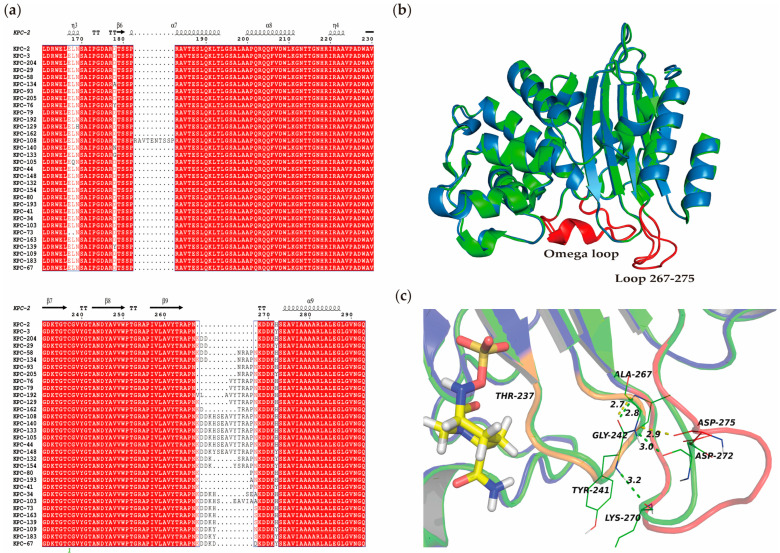
The secondary structure of KPC-204 compared with closely related KPC variants and the stereoscopic structure alignment of KPC-204 with KPC-2. (**a**) The alignment of secondary structures. Closely related KPC variants are those with insertions at Ambler position 269, available in the NCBI database (April 2024). The image shows the Omega loop (residues 164–179) and loop 267–275 regions. Secondary structure elements, α helixes, β sheets, and 310-helixes (represented by η), are indicated. β-strands are rendered as arrows, and strict β-turns are shown as TT letters. (**b**) Stereoscopic structure alignment. KPC-204 and KPC-2 are colored in blue and green, respectively. The Omega loop and loop 267–275 of both KPCs are highlighted in red. (**c**) The comparison of key hydrogen bonds in loop regions 237–243 and 267–275 between KPC-2 and KPC-204. In KPC-2, Tyr241 and Lys270 form a hydrogen bond with a bond length of 3.2 Å, Gly242 and Asp272 form a hydrogen bond with a bond length of 3.0 Å, and Tyr241 and Ala267 form a hydrogen bond with a bond length of 2.8 Å. In KPC-204, the insertion of three amino acids (DDK) at position 269 extends the loop 267–275 range, resulting in a hydrogen bond between Gly242 and Asp275 with a bond length of 2.9 Å and a hydrogen bond between Tyr241 and Ala267 with a bond length of 2.7 Å. The hydrogen bond between Tyr241 and Lys270 is no longer present. KPC-204 and KPC-2 are colored in blue and green, respectively. The loop 237–243 and loop 267–275 of KPC-204 are highlighted in brown and pink, respectively. Hydrogen bonds are depicted as green dashed lines with bond lengths indicated in Å in KPC-2, and yellow dashed lines in KPC-204.

The secondary structures of KPC-204 and other KPC variants with insertions at Ambler position 269 were aligned using data from the NCBI database (April 2024) (Figure 3a). This alignment focused on the Omega loop (residues 164–179) and loop 267–275 regions, both crucial for enzyme activity and antibiotic resistance. Notably, KPC-204 and KPC-29 share an identical KDD insertion at position 269 despite their distinct evolutionary origins (KPC-2 and KPC-3, respectively), indicating potential convergent evolution. KPC-67 contains a KDDKDD insertion at the same position. KPC-29 and KPC-67 were previously reported to exhibit resistance to both CZA and MEM [36], warranting further studies on their enzyme kinetics and structure.

Stereoscopic structural analysis between KPC-204 and KPC-2 reveals a high degree of similarity in the Omega loop (residues 164–179). However, significant divergence is observed in loop 267–275 (Figure 3b). In KPC-204, the insertion of three amino acids (DDK) at position 269 extends the loop 267–275 range.

A comparison of key hydrogen bonds in loop regions 237–243 and 267–275 between KPC-2 and KPC-204 shows that in KPC-2, Tyr241 and Lys270 form a hydrogen bond with a bond length of 3.2 Å, Gly242 and Asp272 form a hydrogen bond with a bond length of 3.0 Å, and Tyr241 and Ala267 form a hydrogen bond with a bond length of 2.8 Å. In KPC-204, Gly242 and Asp275 form a hydrogen bond with a bond length of 2.9 Å, and Tyr241 and Ala267 form a hydrogen bond with a bond length of 2.7 Å. The hydrogen bond between Tyr241 and Lys270 is no longer present (Figure 3c).

### 3.8. Molecular Docking of KPC-204 and KPC-2 with Ceftazidime, Avibactam and Meropenem

**Figure 4:**
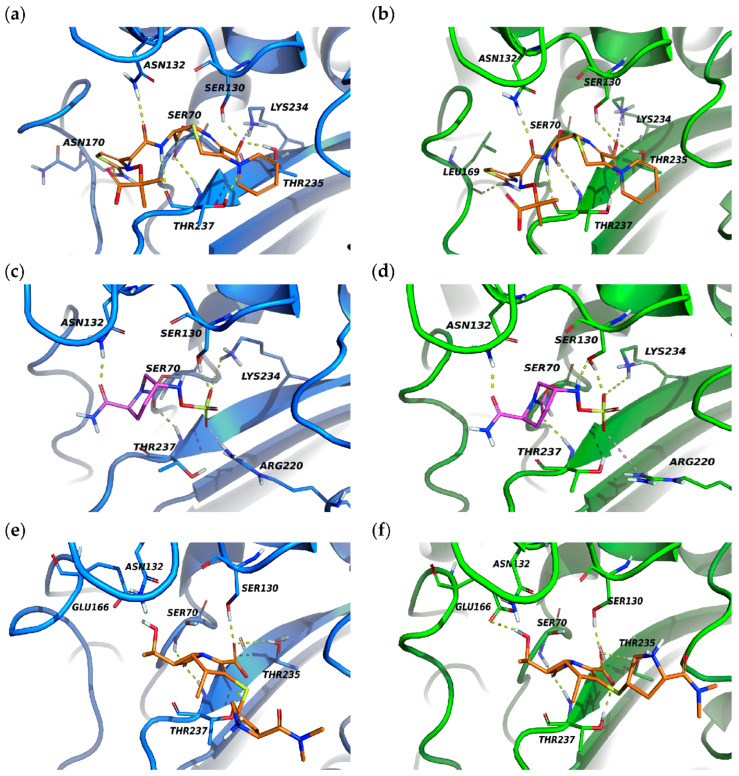
Molecular docking analysis of KPC-204 and KPC-2 interactions with ceftazidime and avibactam. KPC-204 and KPC-2 are colored in blue and green, respectively. Amino acids forming polar bonds with the substrate are shown. (**a**) KPC-204 with ceftazidime: docking score = −11.62 kcal/mol; H-bonds with Asn170, Asn132, Thr237, Thr235, Ser130; and salt bridge with Lys234. (**b**) KPC-2 with ceftazidime: docking score = −11.26 kcal/mol; H-bonds with Leu169, Asn132, Thr237, Thr235, Ser130; and salt bridge with Lys234. (**c**) KPC-204 with avibactam: docking score = −5.09 kcal/mol; H-bonds with Asn132, Thr237, Ser130, Lys234; and salt bridge with Arg220. (**d**) KPC-2 with avibactam: docking score = −8.92 kcal/mol; H-bonds with Asn132, Thr237, Thr235, Ser130, Lys234; and salt bridge with Arg220. (**e**) KPC-204 with meropenem: docking score = −6.33 kcal/mol and H-bonds with Ser130 Thr235 Thr237. (**f**) KPC-2 with meropenem: docking score = −8.72 kcal/mol and H-bonds with Glu166 Ser130 Thr235 Thr237.

We then performed molecular docking simulations to visualize the interactions between KPC-204 and KPC-2 enzymes with ceftazidime, avibactam and meropenem (Figure 4). Interestingly, both KPC-204 and KPC-2 display similar binding scores for ceftazidime (−11.26 kcal/mol and −11.62 kcal/mol, respectively), with slight variations in the specific hydrogen bonding residues (Asn170 vs. Leu169). In contrast, avibactam lacks the hydrogen bond with the side chain of Thr235 in KPC-204, which is present in KPC-2. Moreover, the binding free energy is significantly weaker in KPC-204 (−5.09 kcal/mol) compared to KPC-2 (−8.92 kcal/mol), indicating a reduced affinity for the protein. This diminished interaction may contribute to the resistance observed in KPC-204. KPC-204’s KDD insertion at position 269 likely weakens its meropenem resistance compared to KPC-2. This is suggested by both the weaker binding affinity observed in docking analysis (−6.33 kcal/mol vs. −8.72 kcal/mol) and the absence of a key hydrogen bond with Glu166, which is present in KPC-2.

### 3.9. bla_KPC-204_ Was Located in a Self-Transmissible Plasmid

The transfer frequencies of plasmids pKPC2_015093 and pKPC204_130125 to *E. coli* J53 AziR were 2.4 × 10^−5^ and 8.6 × 10^−4^, respectively, based on the ratio of transconjugants to recipients, highlighting that pKPC204_130125 is readily self-transmissible. MICs for meropenem in *E. coli* J53 AziR carrying the respective plasmids were 32 mg/L and 64 mg/L. MICs for CZA were 0.5 mg/L for J53 with pKPC2_015093 and 64 mg/L for J53 with pKPC204_130125 (Table 1).

## 4. Discussion

Carbapenem antibiotics are the last-resort drugs for treating infections caused by multidrug-resistant Gram-negative bacteria, and CZA is crucial for treating CRKP infections. KPC-2 and KPC-3 are the most prevalent KPC enzymes [4]. KPC-2-producing ST11-type CRKP strains have emerged as a prevalent clonal lineage in China, posing significant clinical challenges [37,38]. With the global utilization of CZA, resistance to CZA has increased, primarily due to novel mutations in the genes encoding the KPC enzyme [4]. Reports of CZA-resistant KPC variants are common. 194 KPC variants were been identified in the NCBI Reference Sequences (RefSeq) database (https://www.ncbi.nlm.nih.gov/pathogens/refgene/#KPC; accessed on 28 April 2024).

Simultaneous resistance to CZA and MEM is rare. Muresu et al. reported [39] a *K. pneumoniae* strain harboring *bla*_KPC-31_ and *bla*_OXA-181_ that was resistant to both CZA and MEM, but the cloning of *bla*_KPC-31_ [40] and *bla*_OXA-181_ [41] did not show MEM resistance. Arcari et al. reported [42] a *K. pneumoniae* strain carrying *bla*_KPC-154_ that showed MEM resistance, but resistance was not observed when the *bla*_KPC-154_ gene was cloned. This suggests the involvement of *bla*_KPC_ gene over-expression and porin modifications, reflecting the complexity of resistance mechanisms.

We identified an ST11-type *K. pneumoniae* strain simultaneously resistant to CZA and MEM, with no prior exposure to CZA. Compared to the control strain 015093, there were no significant differences in *bla*_KPC_ gene expression levels in strain 130125. Our study identified KPC-204, featuring a KDD insertion at position 269 within loop 267–275, which demonstrated resistance to both CZA and MEM after cloning. Notably, instances of CZA resistance in *K. pneumoniae* have been reported even in the absence of prior CZA exposure [36,39,43]. Given the high genomic similarity between this variant and the ST11 KPC2 strain 015093, it suggests a single case arising from a spontaneous mutation.

The amino acid loop 267–275 in the KPC enzyme is a key mutation hotspot. Notably, mutations involving insertions at Ambler position 269 have been identified in several KPC variants, including KPC-204, -29, -58, -134, -93, -205, -76, -79, -192, -129, -162, -108, -140, -133, -105, -44, -148, -132, -154, -80, -193, -41, -34, -103, -73, -163, -139, -109, -183, and -67 (Table S2). The alignment of these KPC variants, including the Omega loop (residues 164–179) and loop 266–275, is shown in Figure 3. Among these, KPC-29, -93, -76, -44, -154, -41, and -67 are associated with resistance to CZA [10,36,42,44,45,46]. Additionally, variants KPC-29 and -154 have also been reported to exhibit resistance to both CZA and MEM [36].

Enzyme kinetic analyses revealed that KPC-204 maintains similar catalytic activity towards ceftazidime and meropenem compared to KPC-2. However, KPC-204 displays a significantly reduced affinity for avibactam, reflected in a 16-fold increase in the IC50 value. Docking simulations support these findings, revealing a weaker interaction between KPC-204 and avibactam compared to KPC-2. The DDK insertion at position 269 of the KPC-204 is situated in the loop 267–275 region, which is peripheral and does not directly interact with the binding pocket or the substrate. KPC-204 may alter the conformation of the binding pocket and substrate affinity by modifying loop 267–275, subsequently affecting loop 237–243.

The KPC-type carbapenemase gene frequently resides on self-conjugative plasmids, facilitating its spread across bacterial populations [4,47,48,49]. Specifically, *bla*_KPC-204_ is harbored on an IncFII/IncR plasmid, known for its capability to transfer horizontally via conjugation, thus highlighting the need for rigorous monitoring. The genetic context of *bla*_KPC-204_ and plasmid pKPC2_015093 shows a high degree of similarity, positioned within a composite transposon, flanked downstream by IS*Kpn27* and upstream by IS*Kpn6*. This arrangement is similar to that found in pKP048 from *K. pneumoniae* isolates in China, yet it diverges from Tn*4401* [50,51]. Additionally, *bla*_KPC-204_, together with *bla*_CTX-M-65_, forms part of a 10 kb integrative composite transposon, enclosed by IS*26* sequences, indicating the potential for mobility and spread that warrants heightened attention. On a positive note, recent studies have demonstrated the efficacy of novel inhibitor combinations, such as imipenem–relebactam and meropenem–vaborbactam, in addressing these resistant strains.

## 5. Conclusions

In conclusion, our study is notable for several reasons. Firstly, we reported an ST11-type clinical CRKP isolate that produces KPC-204, a novel plasmid-borne KPC variant that confers CZA resistance. Secondly, we documented a rare antimicrobial resistance profile, demonstrating resistance to both CZA and meropenem. Thirdly, we investigated the enzymatic changes induced by the KDD insertion at position 269, which diminishes the inhibitory efficacy of avibactam, leading to resistance. Docking simulations support these findings, revealing a weaker interaction between KPC-204 and avibactam compared to KPC-2. Lastly, we examined the genetic context of KPC-204, located on a highly transmissible IncFII/IncR plasmid within a composite transposon, presenting a potential for mobility and spread that warrants significant attention. These findings emphasize the need for the vigilant monitoring and development of novel therapeutic strategies to manage such resistant bacterial strains effectively.

## Figures and Tables

**Table 1:** MIC (mg/L) of antimicrobial agents for isolate 130125, 015093 and *E. coli* DH5α expressing KPC-204 or KPC-2.

	MICs (mg/L) ^a^											
Strains	PIP	TZP	FOX	FEP	ATM	CAZ	CZA	IPM	IMR	MEM	MEV	ETP
130125	>512	256	>512	>512	512	>512	256	64	0.25	64	0.5	64
015093	>512	>512	>512	>512	512	>512	0.5	128	0.25	256	0.06	64
DH5α::pEKPC-2	>512	>512	>512	>512	512	128	0.5	16	0.125	8	0.03	8
DH5α::pEKPC-204	>512	256	>512	512	256	128	64	16	0.125	16	0.125	8
DH5α::pET28a	1	1	2	0.06	0.125	0.25	0.25	0.25	0.06	≤0.015	≤0.015	≤0.015
*E. coli* J53	1	1	1	0.06	0.125	0.25	0.125	0.125	0.06	≤0.015	≤0.015	≤0.015
J53::pKPC2_015093	>512	>512	>512	512	512	512	0.5	32	0.25	32	0.03	32
J53::KPC204_130125	>512	256	>512	512	512	512	64	32	0.25	64	0.25	32

^a^ Abbreviations: PIP, piperacillin; TZP, piperacillin–tazobactam; FOX, cefoxitin; FEP, cefepime; CAZ, ceftazidime; CZA, ceftazidime–avibactam; ATM, aztreonam; IPM, imipenem; IMR, imipenem–relebactam; MEM, meropenem; MEV, meropenem–vaborbactam; ETP, ertapenem. Avibactam was added at a fixed concentration of 4 mg/L, tazobactam at 4 mg/L, relebactam at 4 mg/L, and vaborbactam at 8 mg/L.

**Table 2:** The complete genome and antimicrobial resistant genes of isolate 130125.

	Accession No.	Size, bp	Replicon Type	Resistance Genes	
				β-Lactam	Other
130125_chr	CP148996	5,462,753	-	*bla*_SHV-158_	*aadA2*, *fosA6*
pKPC204_130125	CP148997	154,728	IncR, IncFII	*bla*_KPC-204_, *bla*_TEM-1_, *bla*_CTX-M-65_	*rmtB1*
p1_130125	CP148998	10,060	ColRNAI		
p2_130125	CP148999	5596	-		

**Table 3:** Kinetic parameters of purified β-lactamases KPC-2 and KPC-204 ^a^.

	KPC-2			KPC-204		
β-Lactam	*K_m_* (μM)	*k_cat_* (s^−1^)	*k_cat_*/*K_m_* (μM^−1^·s^−1^)	*K_m_* (μM)	*k_cat_* (s^−1^)	*k_cat_*/*K_m_* (μM^−1^·s^−1^)
Nitrocefin	22.124	97.589	4.411	31.178	116.419	3.734
Ceftazidime	870.413	5.226	0.006	975.154	7.801	0.008
Meropenem	15.283	5.194	0.34	14.157	8.325	0.588

^a^ Data are the means of three independent experiments. Standard deviations were within 15% of the mean value.

**Table 4:** IC_50_ of β-lactamases inhibitors against KPC-2 and KPC-204 ^a^.

	IC_50_ (μM)	
Inhibitor	KPC-2	KPC-204
Avibactam	0.045	0.569
Tazobactam	1.782	0.083
Clavulanic acid	0.887	0.124

^a^ Data are the means of three independent experiments. Standard deviations were within 15% of the mean value.

## Data Availability

The sequence of KPC-204 has been deposited in the NCBI database under GenBank accession number OR979533. The accession numbers for strain 130125 range from CP148996 to CP148999. The accession number for pKPC2_015093 is CP036301.

## Supplementary Materials

The following supporting information can be downloaded at: https://www.mdpi.com/article/10.3390/microorganisms12061193/s1, Table S1: Primers used in this study. Table S2: List of KPC alleles with insertions at Ambler position 269, available in the NCBI database (April 2024). References [4,10,20,26,28,29,30,52,53,54] are cited in the Supplementary Materials.
