# Supplementary material for: Identification of a Novel KPC Variant, KPC-204, Conferring Resistance to Both Carbapenems and Ceftazidime–Avibactam in an ST11 Klebsiella pneumoniae Strain
Source: Microorganisms. 2024 Jun 13;12(6):1193. doi: 10.3390/microorganisms12061193 (PMC11205768; doi:10.3390/microorganisms12061193)
Supplement: Supplementary file 1 [file microorganisms-12-01193-s001.zip › microorganisms-3040152-supplementary.pdf]

Supplemental materials

Table S1. Primers used in this study.

Table S2. List of KPC alleles with insertions at Ambler position 269, available in the NCBI database (April 2024).

Table S1. Primers used in this study.

Primers[a]	Sequence (5′-3′)	Use for
q_KPC_F	GATTGGCTAAAGGGAAACACG	qRT-PCR
q_KPC_R	GCCATACACTCCGCAGGTT	
q_rpoB_F	AATTCCGAGCTGCAATACGTC	qRT-PCR
q_rpoB_R	TTCGCGCTCGTAGATCACCA	
KPC_NdeI_F	AAAAA<u>CATATG</u>GTCATCCGCAGACCAACG	*bla*$_{kpc}$ cloning
KPC_EcoRI_R	CCG<u>GAATTC</u>CAGACTCCTAGCCTAAATGTGA	
KPC-F	GACACACCCATCCGTTAC	*bla*$_{kpc}$ detection
KPC-R	CCAACTCCTTCAGCAACA	
J53_F	ACGGACTAACAGCCTGGAAA	confirming J53 for
J53_R	TAGCGTATCCAGCGTCACTT	conjugation
T7	TAATACGACTCACTATAGGG	confirming cloning
T7ter	TGCTAGTTATTGCTCAGCGG	fragment
IncFII_F	AGAAGGAGTGAGCACAGA	replicon confirming
IncFII_R	TTGGCGAGTCAGAAAGATT	
KPC_frg_F	TGTATTTTCAGGGTGCTAGCCTGACCAACCTCGTCGCG	*bla*$_{KPC}$ gene sequence
KPC_frg_R	TGGTGGTGGTGGTGCTCGAGTTACTGCCCGTTGACGCC	(residues 25-293)

KPC_vec_F	CTCGAGCACCACCACCACC	the pET-28a vector
KPC_vec_R	GCTAGCACCCTGAAAATACAGATTT	

[a]All Primers except the universal T7 and T7ter were self-designed. Restriction sites are underlined.

Table S2. List of KPC alleles with insertions at Ambler position 269, available in the NCBI database (April 2024).

KPC-variant[a]	Accession Number	Divergence from	Omega loop	Loop 266-275	CZA treated	MICs (mg/L)[b]		PMID	References
						CZA	MEM		
KPC-204	OR979533	KPC-2		ins_269_KDD	No	128	16		
KPC-29	AY034847	KPC-3		ins_269_KDD	Yes	24	16	34339281	[1]
KPC-58*	MT463289	KPC-2		ins_269_KDDNRAPN					
KPC-134*	OP293349	KPC-2	D179A	ins_269_KDDNRAPN					
KPC-93	MZ569034	KPC-2		ins_269_NRAPN	Yes	64	0.06	35416703	[2]
KPC-205*	JAYEEW010000028	KPC-3		ins_269_NRAPN					
KPC-76	MT550690	KPC-2	D179Y	ins_269_VYTRAPN	Yes	16	≤0.03	34935416,35588280	[3,4]
KPC-79	EU729727	KPC-2		ins_269_VYTRAPN	Yes	4	≤0.03	34935416	[4]
KPC-192*	OR529436	KPC-2		ins_269_VLAVYTRAPN					
KPC-129*	ON751738	KPC-2	N170H	ins_269_KVYTRAPN					
KPC-162*	OQ579138	KPC-2		ins_269_KDTRAPN					
KPC-108	GQ140348	KPC-2		ins_269_KDDKHSEAVYTRAPN	Not reported	NA	NA	35980232	[5]
KPC-140*	OP503888	KPC-2	D179N	ins_269_KDDKHSEAVYTRAPN					
KPC-133	OP081531	KPC-2	D179G	ins_269_KDDKHSEAVYTRAPN	Not reported	NA	NA	27242772	[6]
KPC-105*	GQ140348	KPC-2	L169Q	ins_269_KDDKHSEAVYTRAPN					

KPC-44	MK823188	KPC-2		ins_269_KDDKHSEAVYTRAPN	Yes	＞16	16	31088601	[7]
KPC-148*	JAOZYA010000028	KPC-3		ins_269_KDDKYSEAVYTRAPN					
KPC-132*	OP081092	KPC-3		ins_269_KDDKSRAP					
KPC-154	OQ096263	KPC-3		ins_269_KDDKYSRAP	Yes	16	16	37877547	[8]
KPC-80*	MW444845	KPC-2		ins_269_KPN					
KPC-193*	OR568565	KPC-2		ins_269_KAN					
KPC-41	MK497255	KPC-3		ins_269_KPN	Yes	128	0.5	31527032	[9]
KPC-34	KU985429	KPC-2		ins_269_KDDKHSEA	Not reported	NA	NA	29855588	[10]
KPC-103*	GQ140348	KPC-2		ins_269_KDDKHSEAVIAA					
KPC-73*	MT833886	KPC-2	del_168-169_EL	ins_269_KDDKHS					
KPC-163*	OQ579139	KPC-2		ins_269_KDDKHS					
KPC-139*	HQ342889	KPC-2	D179Y	ins_269_KDDKHS					
KPC-109*	GQ140348	KPC-3		ins_269_KDDKYN					
KPC-183*	OR282800	KPC-3		ins_269_KDDKYS					
KPC-67	MT809697	KPC-3		ins_269_KDDKDD	No	≥24	≥8	34339281	[1]

[a]Variants indicated with a star correspond to variants unpublished. The KPC-154 clinical strain showed MEM resistance (16 mg/L), but when cloned into the pCR-Blunt II TOPO-NeoR Vector, it did not exhibit resistance (≤0.12 mg/L). KPC-44 was only reported to have MEM resistance (16 mg/L) in the clinical strain without cloning experiment results.
[b]Abbreviations: CZA, Ceftazidime-avibactam; MEM, Meropenem; NA : not applicable.

1. Carattoli, A.; Arcari, G.; Bibbolino, G.; Sacco, F.; Tomolillo, D.; Di Lella, F.M.; Trancassini, M.; Faino, L.; Venditti, M.; Antonelli, G.; et al. Evolutionary Trajectories toward Ceftazidime-Avibactam Resistance in Klebsiella Pneumoniae Clinical Isolates. Antimicrob Agents Chemother 2021, 65, e0057421, doi:10.1128/AAC.00574-21.
2. Wu, Y.; Yang, X.; Liu, C.; Zhang, Y.; Cheung, Y.C.; Wai Chi Chan, E.; Chen, S.; Zhang, R. Identification of a KPC Variant Conferring Resistance to

Ceftazidime-Avibactam from ST11 Carbapenem-Resistant Klebsiella Pneumoniae Strains. Microbiol Spectr 2022, 10, e0265521, doi:10.1128/spectrum.02655-21.

3. Ding, L.; Shi, Q.; Han, R.; Yin, D.; Wu, S.; Yang, Y.; Guo, Y.; Zhu, D.; Hu, F. Comparison of Four Carbapenemase Detection Methods for blaKPC-2 Variants. Microbiol Spectr 2021, 9, e0095421, doi:10.1128/Spectrum.00954-21.
4. Shi, Q.; Han, R.; Guo, Y.; Yang, Y.; Wu, S.; Ding, L.; Zhang, R.; Yin, D.; Hu, F. Multiple Novel Ceftazidime-Avibactam-Resistant Variants of blaKPC-2-Positive Klebsiella Pneumoniae in Two Patients. Microbiol Spectr 2022, 10, e0171421, doi:10.1128/spectrum.01714-21.
5. Hobson, C.A.; Pierrat, G.; Tenaillon, O.; Bonacorsi, S.; Bercot, B.; Jaouen, E.; Jacquier, H.; Birgy, A. Klebsiella Pneumoniae Carbapenemase Variants Resistant to Ceftazidime-Avibactam: An Evolutionary Overview. Antimicrob Agents Chemother 2022, 66, e0044722, doi:10.1128/aac.00447-22.
6. Scandorieiro, S.; de Camargo, L.C.; Lancheros, C.A.C.; Yamada-Ogatta, S.F.; Nakamura, C.V.; de Oliveira, A.G.; Andrade, C.G.T.J.; Duran, N.; Nakazato, G.; Kobayashi, R.K.T. Synergistic and Additive Effect of Oregano Essential Oil and Biological Silver Nanoparticles against Multidrug-Resistant Bacterial Strains. Front Microbiol 2016, 7, 760, doi:10.3389/fmicb.2016.00760.
7. Räisänen, K.; Koivula, I.; Ilmavirta, H.; Puranen, S.; Kallonen, T.; Lyytikäinen, O.; Jalava, J. Emergence of Ceftazidime-Avibactam-Resistant Klebsiella Pneumoniae during Treatment, Finland, December 2018. Euro Surveill 2019, 24, 1900256, doi:10.2807/1560-7917.ES.2019.24.19.1900256.
8. Arcari, G.; Cecilia, F.; Oliva, A.; Polani, R.; Raponi, G.; Sacco, F.; De Francesco, A.; Pugliese, F.; Carattoli, A. Genotypic Evolution of Klebsiella Pneumoniae Sequence Type 512 during Ceftazidime/Avibactam, Meropenem/Vaborbactam, and Cefiderocol Treatment, Italy. Emerg Infect Dis 2023, 29, 2266–2274, doi:10.3201/eid2911.230921.
9. Mueller, L.; Masseron, A.; Prod'Hom, G.; Galperine, T.; Greub, G.; Poirel, L.; Nordmann, P. Phenotypic, Biochemical, and Genetic Analysis of KPC-41, a KPC-3 Variant Conferring Resistance to Ceftazidime-Avibactam and Exhibiting Reduced Carbapenemase Activity. Antimicrobial Agents and Chemotherapy 2019, 63, 10.1128/aac.01111-19, doi:10.1128/aac.01111-19.
10. Chiu, S.-K.; Ma, L.; Chan, M.-C.; Lin, Y.-T.; Fung, C.-P.; Wu, T.-L.; Chuang, Y.-C.; Lu, P.-L.; Wang, J.-T.; Lin, J.-C.; et al. Carbapenem Nonsusceptible Klebsiella Pneumoniae in Taiwan: Dissemination and Increasing Resistance of Carbapenemase Producers During 2012-2015. Sci Rep 2018, 8, 8468, doi:10.1038/s41598-018-26691-z.
